# Alterations of Suckling Piglet Jejunal Microbiota Due to Infection With Porcine Epidemic Diarrhea Virus and Protection Against Infection by *Lactobacillus salivarius*

**DOI:** 10.3389/fvets.2021.771411

**Published:** 2021-12-09

**Authors:** Wanting Dong, Ning Ding, Yu Zhang, Zhen Tan, Xiangdong Ding, Qin Zhang, Li Jiang

**Affiliations:** ^1^Key Laboratory of Animal Genetics, Breeding and Reproduction, Ministry of Agriculture and National Engineering Laboratory for Animal Breeding, College of Animal Science and Technology, China Agricultural University, Beijing, China; ^2^College of Animal Science and Technology, Hainan University, Haikou, China; ^3^Shandong Provincial Key Laboratory of Animal Biotechnology and Disease Control and Prevention, Shandong Agricultural University, Tai'an, China

**Keywords:** porcine epidemic diarrhea virus, jejunal microbiota, *Lactobacillus salivarius*, suckling piglets, 16S rRNA

## Abstract

The high mortality of neonatal piglets due to porcine epidemic diarrhea virus (PEDV) infection has caused huge economic losses to the pig industry. The intestinal microbiota is an important barrier against invaders entering the gastrointestinal route. In this study, we examined the differences between intestinal microbiota of PEDV-infected and healthy piglets. According to the viral copy numbers, 16 crossbred (Landrace-Yorkshire) piglets were divided into three groups: uninfected, low virus load, and high virus load groups. Next, 16S rRNA sequencing was performed to determine the microbiota composition in jejunal content and jejunal mucosal samples from the three groups. PEDV infection induced an imbalance in the microbiota of both jejunal content and jejunal mucosa. The abundance of phylum Firmicutes was higher in uninfected piglets than in infected piglets, whereas the abundance of Proteobacteria was lower in uninfected piglets. Principal coordinate analysis showed significant separation of jejunal microbiota between different groups. Linear discriminant analysis (LDA) effect size (LEfSe) identified *Lactobacillus salivarius* as a potential biomarker among three groups at the level of species. Then, *in vitro, L. salivarius* was able to suppress the infection of PEDV to IPEC-J2 cells and decreased the expression of *GRP78* (Glucose-regulating protein 78). In addition, we detected the mRNA expression of genes involved in the FAK/PI3K/Akt signaling pathway. When IPEC-J2 cells were treated with *L. salivarius* before PEDV infection, the mRNA expression levels of *ITGA1, ITGA5, ITGB5, FAK, PIK3R1, PIK3CA* and *AKT1* were significantly higher than those in the control cells (without treatment) at different times post-infection, indicating that *L. salivarius* may upregulate the FAK/PI3K/Akt signaling pathway in IPEC-J2 cells to resist PEDV infection. In summary, PEDV infection altered microbial communities in both jejunal content and jejunal mucosa. *L. salivarius* has a protective effect against PEDV infection in IPEC-J2 cells. This study provides a potentially effective strategy to prevent the occurrence and control the spread of PED in the pig production.

## Introduction

Porcine epidemic diarrhea (PED) is caused by infection with the highly infective porcine epidemic diarrhea virus (PEDV). PED is a fatal gastrointestinal disease among suckling piglets that is characterized by high morbidity and mortality. Once infected by PEDV, most sucking piglets suffer severe vomiting and watery diarrhea (1). The rapid spread of PEDV has caused huge economic losses in the pig industry worldwide. Since 2010, PEDV outbreaks have been widely reported in Asia and North America (2, 3). For example, approximately 1,000,000 piglets died from PEDV outbreaks in southern China in the winter of 2010 (4). In the United States, economic loss attributed to PEDV infection accounted for over 10% of the total losses in the pig industry from 2013 to 2014 (5). PED has also broken out in Canada, Korea, and other countries (6–8).

PEDV is an enveloped, single-stranded positive sense RNA virus that belongs to the *Coronaviridae* family (9). It encodes four structural proteins including the spike (S), envelope (E), membrane (M), and nucleocapsid (N) proteins, and an accessory protein. Due to the high variability of the PEDV genome, particularly the portion encoding the S protein (10, 11), current vaccines cannot provide proper and effective protection to piglets. Therefore, an essential approach to prevent PEDV infection must be developed.

A disturbed gut microbiome contributes to the development of gastrointestinal diseases (12). The commensal gut microbiota is a mediator between the host immune system and pathogens (13, 14). Increasing evidence has shown that gut commensal microbiota could help prevent pathogenic invasion by competing for receptors and enteric nutrients, stimulating the innate immune system, producing antimicrobial compounds, and creating an anti-pathogen microenvironment (15, 16). In addition, gut microorganisms play an important role in the immune system (17–19).

16S rRNA gene sequencing has been widely used to understand the effects of PEDV infection one microbiota composition (20–25). However, the differences in sampling sites and limitations of the functional analyses in these studies have hindered the identification of bacteria associated with PEDV infection. Moreover, the functional gut microbiota that impacts PEDV infection remains unknown.

In this study, we hypothesized that the regulation of the host innate immune system by the microbiota may help prevent PEDV infection. To test this hypothesis, we characterized and compared the jejunal mucosa- and jejunal content-associated microbiota of naturally PEDV-infected and healthy piglets, using 16S rRNA gene sequencing. Based on the differences in microbiota composition found between infected and uninfected piglet, we then evaluated the anti-PEDV potential of *Lactobacillus salivarius in vitro*. Our findings demonstrate the potential for using jejunal microbiota to improve piglet resistance to PEDV infection.

## Materials and Methods

### Ethics Statement

This work was conducted in accordance with the guidelines approved by the Quality Supervision, Inspection, and Quarantine of the People's Republic of China (GB/T 17236–2008). The animal experimental proposals were approved by the Animal Welfare Committee of China Agricultural University (permit number: DK996) and performed in accordance with the Guidelines for Experimental Animals of the Ministry of Science and Technology (Beijing, China).

### Animals and Samples

The piglets in this study came from a commercial breeding farm in Shandong Province (Binzhou, China). All the piglets were offspring of Landrace and Yorkshire pigs. The sows were fed corn and soybean meal based commercial diets under the same conditions. When the sows gave birth within a week, multiple piglets had diarrhea symptoms in farrowing house. Four litters of adjacent sows were chosen that gave birth at similar times in the farrowing house with diarrhea symptoms, and two piglets with diarrhea symptoms and two piglets without diarrhea symptoms in each litter were chosen. A total of 16 piglets (Landrace-Yorkshire hybrids) were selected. All piglets were 7–9 days old and weighed about two kilograms during the normal lactation process. The selected suckling piglets were slaughtered, and samples of blood, jejunum chyme and jejunum mucosa were collected from each piglet within 30 min under aseptic conditions. All samples were collected in sterile tubes and snap frozen in liquid nitrogen.

### PEDV Detection

To confirm the status of PEDV infection in each piglet, quantitative real-time PCR (qPCR) was used to detect PEDV. First, total virus RNA was extracted from the jejunal mucosa leach supernatant using the QIAamp viral RNA MINI kit (QIAGEN, Valencia, CA). Reverse transcription was performed using the PrimeScript®RT reagent kit with a gDNA eraser (Takara, Japan). Viral quantification was carried out using the absolute quantification method. To construct the recombinant plasmid, a 170bp fragment of the PEDV CV777 strain was amplified and cloned into the pTZ57R/T vector (2886bp, Takara, Japan). The concentration of recombinant plasmids was measured using Nanodrop 2000 (Thermal Fisher, USA), and the plasmid copy number was measured using the formula: number of copies of PEDV = (concentration in ng ×6.022 ×10^23^) / (genome length ×10^9^ ×650). To generate the standard curve for SYBR Green I real time qPCR, 10-fold serial dilutions of plasmids DNA from 1 ×10^8^copies/μLto 1 ×10^3^ copies/μL were produced. Thereafter, the number of viral copies was determined for these assays. Specific primer sequences (forward: 5′-GGTTCTATTCCCGTTGATGAGGT-3′; reverse: 5′ -AACACAAGAGGCCAAAGTATCCAT-3′) were used for the PEDV CV777 detection, as previously described (26). SYBR Green I real time qPCR was performed using a Roche LightCycler480 instrument with a total reaction volume of 20 μL (comprising 10 μL SYBR-Green I Master Mix, 1 μL upstream primer, 1 μL downstream primer, 1 μL cDNA template, and ddH_2_O) (Roche Applied Science). All qPCR reactions were performed in 96-well plates, and each sample was run in triplicate.

Based on viral copy number and phenotype, piglets were classified into uninfected [U group, *n* = 8, −1.0904 ± 0.2029 log(copies)], low viral load [L group, *n* = 3, 0.3713 ± 0.4093 log(copies)], and high viral load [H group, *n* = 5, 3.0170 ± 0.522 log(copies)]. Both L and H groups were composed of piglets suffering from diarrhea, but their viral load significantly differed (Kruskal–Wallis test, *p* < 0.01) (Supplementary Figure 1).

### 16S rRNA Gene Sequencing and Analysis

Microbial genomic DNA was extracted from jejunal mucosa and jejunal digesta samples and purified using a QIAamp DNA Stool Mini Kit (Qiagen, Hilden, Germany), according to the manufacturer's protocol. Thereafter, the DNA concentration was measured using a UV-Vis spectrophotometer (NanoDrop 2000, Thermo Fisher Scientific, Waltham, MA, USA), and the quality was confirmed with 1% agarose gel electrophoresis.

The V4 region of the 16S rRNA gene from jejunal mucosa samples was PCR-amplified using 515F-806R universal primers combined with adapter and barcode sequences (forward primer: 5′ -GTGCCAGCMGCCGCGGTAA-3′; reverse primer: 5′ -GGACTACHVGGGTWTCTAAT-3′). For jejunal digesta samples, the V3-V4 region of the 16S rRNA gene was amplified with 314F-608R universal primers (forward primer:5′-CCTAYGGGRBGCASCAG-3′;reverse primer: 5′ -GGACTACNNGGGTATCTAAT-3′) (27). The total reaction volume was 30 μL, containing 15 μL Phusion High-Fidelity PCR Master Mix (New England Biolabs, Ipswich, MA, USA), 0.2 μM forward and reverse primers, and 10 ng template DNA. The thermocycling protocol was as follows: 98°C for 1 min, followed by 30 cycles of 98°C for 10 s, 50°C for 30 s, and 72°C for 60 s; and a final extension at 72°C for 5 min. The PCR products were purified using the GeneJET Gel Extraction Kit (Thermo Fisher Scientific) following the manufacturer's protocol. Thereafter, the constructed library was sequenced using the Illumina Hiseq 2500 platform (2 ×250 paired ends). The data were deposited in the National Center for Biotechnology Information's Short Read Archive under the accession number SRP334838.

To minimize the effects of random sequencing errors, raw FASTQ files were demultiplexed, quality-filtered using Trimmomatic (version 0.33), and merged using FLASH version 1.2.7 (28, 29). To obtain high-quality tag sequences, initial base sites with Phred score < 20 were truncated; tags were filtered if their continuous, high-quality base length was less than three-quarters of the whole sequence; chimeric sequences were removed using UCHIME (version 4.2) (30). Sequences with ≥ 97% similarity were assigned to the same operational taxonomic units (OTUs) using USEARCH (version 10.0) (31). A representative sequence for each OTU was screened and taxonomically analyzed against the SILVA database (https://www.arb-silva.de/) using Ribosomal Database Project Classifier (version 2.2) (32). The composition of each sample community was determined at the phylum, class, order, family, genus, and species level.

The alpha diversity indices were evaluated using Mothur (version 1.30) (33). The beta diversity per group was calculated using principal coordinate analysis (PCoA) based on weighted UniFrac distances (34, 35) in QIIME (version 1.9.1). Linear discriminant analysis (LDA) effect size (LEfSe) was performed to identify biomarkers that differed significantly between groups (35). Statistical significance was set at an LDA score of 3.

### Cell Lines, Bacteria Strains and Virus

The porcine intestine epithelial cell J2 (IPEC-J2) cell line was maintained in our laboratory. Vero E6 cells and PEDV CV777 strain were kindly provided by Dr. Jun Han at the College of veterinary Medicine, China Agricultural University (Beijing, China). IPEC-J2 and Vero E6 cell lines were grown in Dulbecco's Modified Eagle Medium (DMEM, Gibco) supplemented with 10% fetal bovine serum (FBS, Gibco) without penicillin-streptomycin. The cells were cultured in a humidified atmosphere at 37°C and 5% CO_2_. The *Lactobacillus salivarius* JCM strain (Genebank: AB289296) was obtained from the China General Microbiological Culture Collection Center (CGMCC). *L. salivarius* cells were grown in MRS broth (Oxoid) at 37°C for 18 h. The number of CFU/mL was measured after incubation at 37°C for 18 h via serial fold dilutions and planting on MRS agar (Oxoid). Vero E6 cells were seeded in 96-well plates, inoculated with the PEDV CV777 strain at a multiplicity of infection (MOI) of 0.1, and cultured in serum-free DMEM for 60 h at 37°C and 5% CO_2_ in a humidified environment. The tilter of the progeny virions was measured using the TCID50 method.

### Establishment of an *in vitro* PEDV Infection Model

To determine the reasonable duration of virus infection, the PEDV CV777 was inoculated into IPEC-J2 cells at four different time points. IPEC-J2 cells were seeded in six-well plates and treated with the PEDV CV777 strain at a MOI of 0.1. After 0.5, 1, 2, and 3 h of incubation for at 37°C and 5% CO_2_, the cells were washed three times with PBS to remove unattached virions. Thereafter, internalized viruses were released through three freeze-thaw cycles and quantified by qPCR, as mentioned above.

After estimating the best time point, we tested whether *L. salivarius* strain can provide protection against PEDV by incubating IPEC-J2 cells with different concentrations of *L. salivarius* before PEDV infection. First, IPEC-J2 cells were seeded in six-well plates and pretreated with either antibiotic-free DMEM or *L. salivarius* at multiplicity of bacteria (MOB) of 1, 10, or 100, respectively. After 2 h of incubation, the unbound *L. salivarius* cells were washed three times with PBS. Thereafter, IPEC-J2 cells were infected with PEDV CV777 strain at a MOI of 0.1. Cells were cultured at 37°C and 5% CO_2_ for 1 h and 2 h, in separate culture vessels. Finally, the unattached virions were removed, and IPEC-J2 cells were washed with PBS three times before being overlaid with 1 mL DMEM. To release the internalized viruses, the cells were subjected to three freeze-thaw cycles. The supernatants were collected and the total numbers of infected cells were quantified via qPCR for absolute quantification. The most appropriate *L. salivarius* dose was determined to be one corresponding to the lowest quantity of PEDV-infected cells.

Based on the system established above, IPEC-J2 cells were seeded in six-well plates and pretreated with either antibiotic-free DMEM or *L. salivarius* at a MOB of 1 for 2 h. Thereafter, unbound bacteria were removed by washed the cells with PBS three times. Subsequently, IPEC-J2 cells were infected with CV777 at a MOI of 0.1 and cultured at 37°C and 5% CO_2_ for 2 h. Then, the expression levels of candidate genes in IPEC-J2 cells were quantified via qPCR at 2, 8, and 16 h post-infection.

### Statistical Analysis

All experiments were performed in triplicates and repeated three times. Data are presented as the means ± standard error. Differences in the PEDV copy number between the groups were analyzed using the Kruskal-Wallis test, followed by Bonferroni *post hoc* adjustment. Differences in the expression levels of genes between the two groups were compared using Student's *t*-test. A *p* value below 0.05 and 0.01 were considered significant and very significant, respectively. Statistical analysis was performed using the R package.

## Results

### Jejunal Microbiota Profile of the Different Groups

As PEDV invades the host *via* the jejunum, we focused on the jejunal microbiota rather than fecal microbiota. In addition, this study aimed to distinguish “transient microbes” from “resident microbes.” Therefore, microbes attached to the jejunal mucosa were sequenced and referred to as jejunal mucosal microbiota. The microbiota in the jejunal digesta, which we referred to as the jejunal content microbiota, was also sequenced. The mean number of taxon tags in jejunal mucosa samples was 92,311, ranging from 54,926 to 126,987 reads, whereas the average of taxon tags of jejunal digesta samples was 90,448, ranging from 61,510 to102,058. The coverage of jejunal mucosa samples and jejunal digesta samples was 98.472 and 99.995%, respectively, indicating reliable sequencing accuracy.

**Figure 1:**
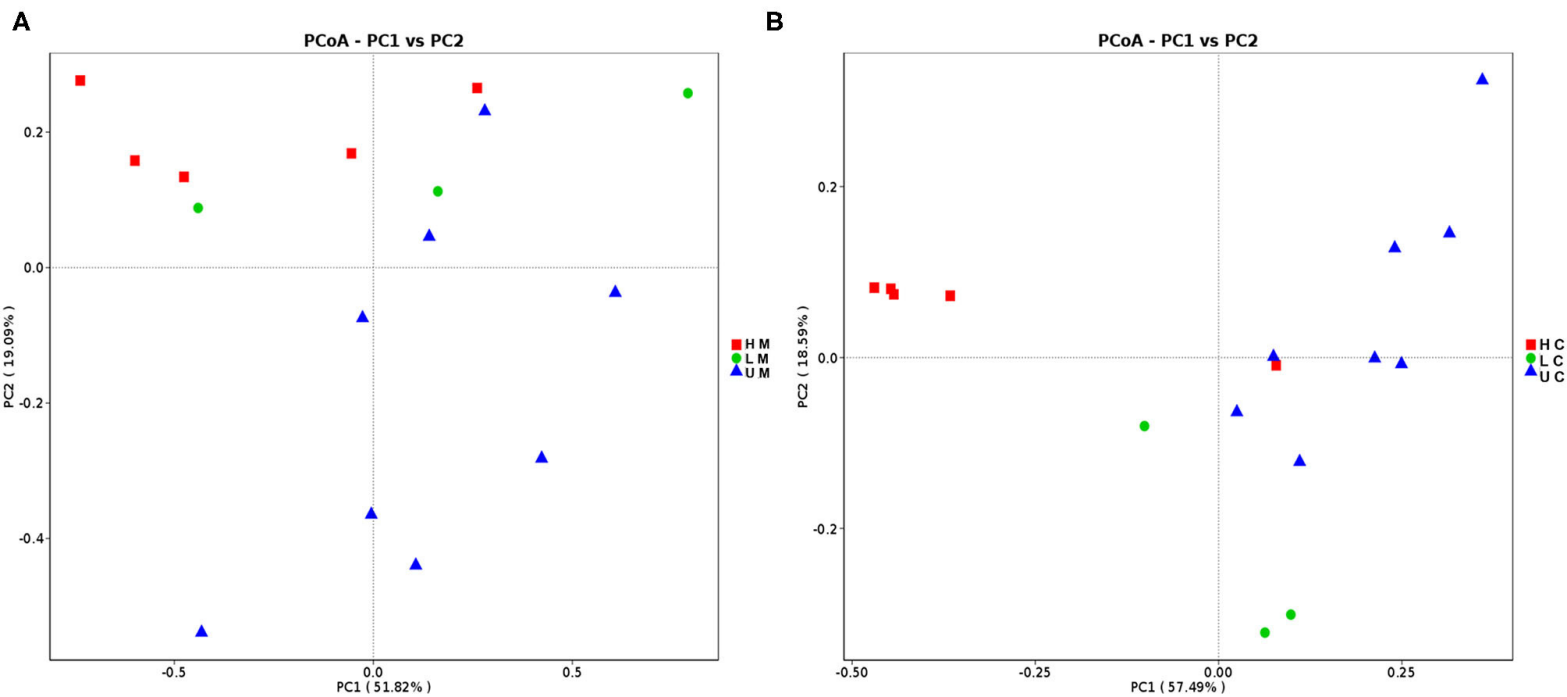
Principal coordinates analysis (PCoA) of jejunal mucosa **(A)** and jejunal content **(B)** microbiome samples from uninfected, low viral load and high viral load groups. HM, jejunal mucosa microbiome samples from the high viral load group; LM, jejunal mucosa microbiome samples from the low viral load group; UM, jejunal mucosa microbiome samples from uninfected group; HC, jejunal content microbiome samples from the high viral load group; LC, jejunal content microbiome samples from the low viral load group; UC, jejunal content microbiome samples from the uninfected group.

Different measures of the alpha diversity index were calculated to characterize the microbial diversity within individuals. Although a different trend of alpha diversity index was observed between piglets with diverse infection status, the difference was not statistically significant (*p* > 0.05). The alpha diversity index of the U group was higher than that of the H group for both sample types (Supplementary Table 1). The PCoA scatterplot revealed clear segregation of gut bacterial communities between piglets in different groups based on beta diversity; however, no clear segregation was observed between different sample types (Figure 1).

**Figure 2:**
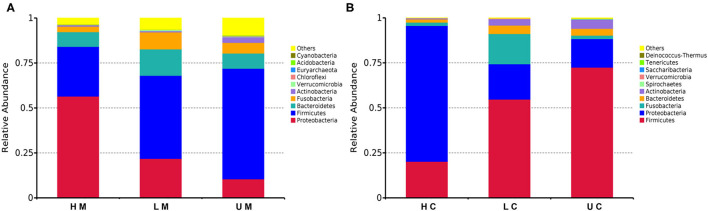
Histogram of the dominant phyla in jejunal mucosa **(A)** and jejunal content **(B)** microbiome samples from the uninfected, low viral load, and high viral load groups.

Thereafter, the taxonomic analysis indicated that four phyla, namely, Firmicutes, Bacteroidetes, Proteobacteria, and Fusobacteria, accounted for 90% of the bacteria in each group (Figure 2). At phylum level, Firmicutes (48.03%), Proteobacteria (26.99%), Bacteroidetes (9.54%), Fusobacteria (5.69%) and Actinobacteria (1.90%) were identified as the dominant phyla in the bacterial communities of all mucosa samples, among which Firmicutes and Proteobacteria accounted for over 65% of the bacteria. The abundance of Firmicutes in the U group was the highest (61.47%), followed by 46.21% in the L group and 27.64% in the H group. The H group had a higher abundance of Proteobacteria (56.47%) than the L group (21.83%) and U group (10.51%), whereas the abundance of Bacteroidetes and Fusobacteria in the L group was higher than that in the H and U group (Figure 2A). In jejunal content samples, the dominant phyla were the same as those of jejunal mucosa, whereas the abundance of Firmicutes (52.83%) and Proteobacteria (35.22%) was higher than that in jejunal mucosa samples (Firmicutes, 48.03%; Proteobacteria, 26.99%) (Figure 2B).

**Figure 3:**
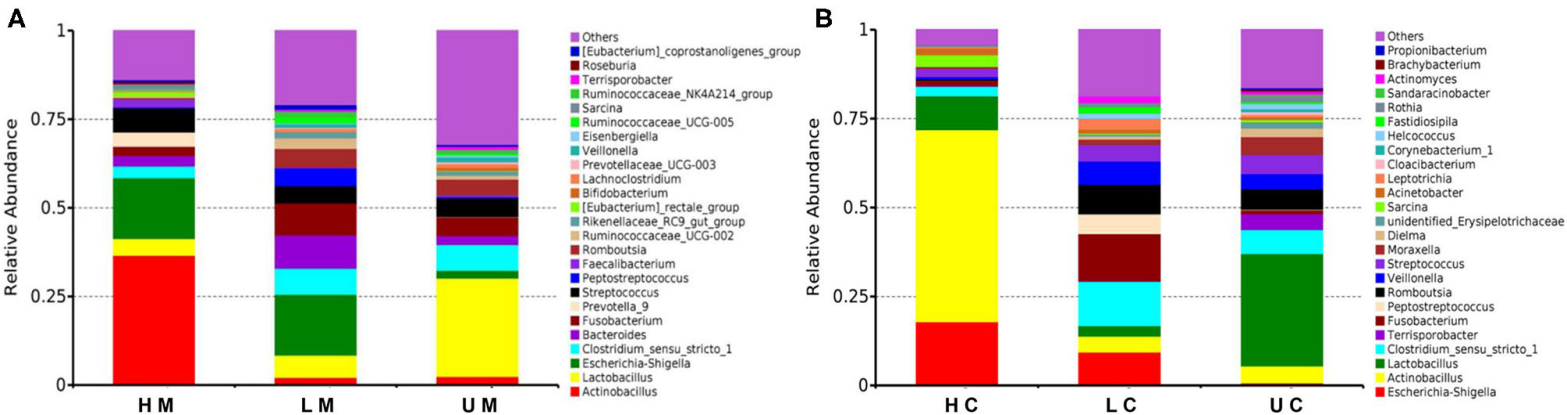
Histogram of the dominant genera in jejunal mucosa **(A)** and jejunal content **(B)** microbiome samples from the uninfected, low viral load, and high viral load groups.

At the genus level, *Actinobacillus, Lactobacillus, Escherichia-Shigella, Clostridium_sensu_stricto_1, Fusobacterium* and *Bacteroides* were the dominant genera in each group in jejunal mucosa (Figure 3A). In the U group, the abundance of *Lactobacillus* was 27.74%, which was higher than that in the L (6.30%) and H groups (4.74%). The H group had a higher abundance of *Actinobacillus* (36.61%) than the U group (2.45%) and the L group (2.15%). In contrast, the L group showed a higher abundance of *Bacteroides* (9.36%) and *Fusobacterium* (9.00%). The abundance of *Escherichia-Shigella* in infected groups (17.18% and 17.14% in the H and L groups, respectively) was higher than that in the U group (2.15% in the U group). In the jejunal contents, *Terrisporobacter* was the dominant microbe (Figure 3B).

**Figure 4:**
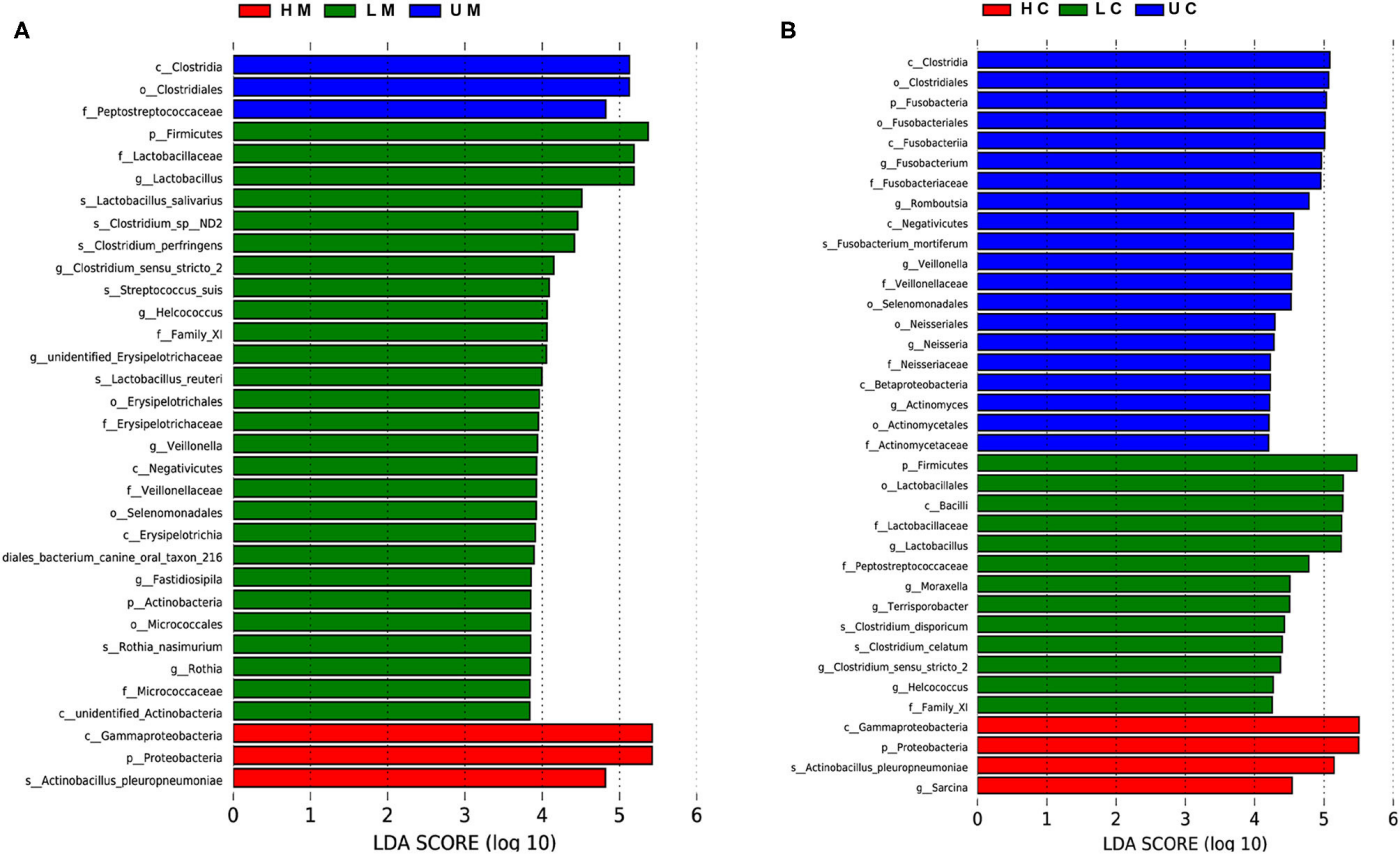
Linear discriminant analysis (LDA) effect size (LEfSe) of jejunal mucosa and content microbiome samples from the uninfected, low viral load, and high viral load groups. Histogram of LDA scores computed for differences in the proportions of bacteria at different taxonomic levels in jejunal mucosa **(A)** and jejunal content **(B)** samples between uninfected, low viral load, and high viral load groups.

LEfSe analysis was performed to identify potential biomarkers distinguishing between groups. In jejunal mucosa samples, 33 terms of differential bacteria belonging to different taxonomic level were found in the three groups (Figure 4A). There were 37 significantly enriched differential bacteria in the jejunal digesta samples (Figure 4B). At the species level, seven significant bacteria were found in jejunal mucosa samples, compared to four significant bacteria in jejunal digesta samples. *Actinobacillus pleuropneumoniae* was enriched in all samples of the infected group. *L. salivarius, Lactobacillus reuteri, Streptococcus suis, Clostridium pernser, Rothia nasimurium* and *Clostridium sp ND2* were significantly enriched in the mucosa of uninfected piglets.

### *L. salivarius* Suppresses PEDV Entry *in vitro*

**Figure 5:**
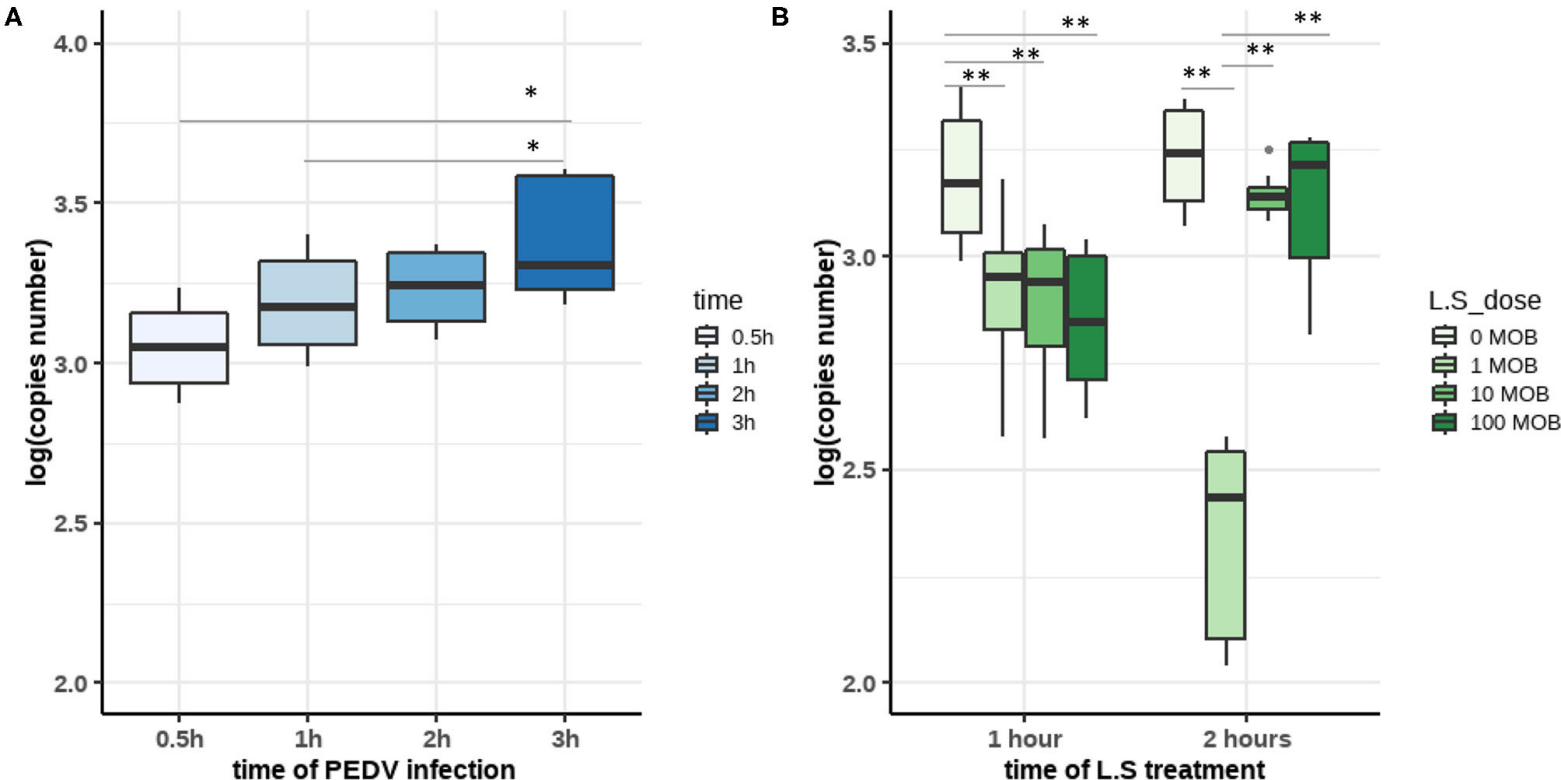
**(A)** The copy numbers of porcine epidemic diarrhea virus (PEDV) in infected IPEC-J2 cells at different time points. IPEC-J2 cells were infected with the PEDV CV777 strain at 0.1 MOI. After 0.5 h, 1 h, 2 h, and 3 h of incubation, viral copies were quantified by qPCR. **(B)** *Lactobacillus salivarius* inhibits the infection of IPEC-J2 cells by PEDV. IPEC-J2 cells were treated with different concentrations of *L. salivarius* (1, 10, and 100 MOI) for 2 h before PEDV infection. Then, IPEC-J2 cells were infected with PEDV CV777 at 0.1 MOI for 1 or 2 h. The copy numbers of PEDV in each group were detected by qPCR. The differences between the groups were analyzed using the Kruskal–Wallis test. Significant differences are marked with asterisks. ^*^: *p* < 0.05, ^**^: *p* < 0.01.

Members of *Lactobacillus* have been regarded as probiotics; therefore, we were particularly interested in determining whether the enrichment of *Lactobacillus spp*. such as *L. salivarius* in uninfected piglets could provide effective protection against PEDV infection. We established an *in vitro* model using IPEC-J2 cells, the PEDV CV777 strain, and the *L. salivarius* JCM strain to simulate different infection statuses. First, virus replication kinetics experiments were performed to determine in time point at which PEDV fully entered the cell. IPEC-J2 cells were inoculated with PEDV at 0.1 MOI and harvested at 0.5, 1, 2, and 3 h post-infection. Our results showed that the number of viral copies in the cells increased with time. The difference in the number of viral RNA copies between cells harvested at 3 h [3.379027 ± 0.1831912 log(copies)] and 2 h [3.234857 ± 0.1096953 log(copies)] post-infection was not significant, whereas the number of viral RNA copies in cells at 0.5 h [3.052131 ± 0.1327514 log(copies)] and 1 h [3.188493 ± 0.1515467 log(copies)] post-infection was significantly lower than that in cells harvested at 3 h post-infection. The results indicated that PEDV entry occurred between 1 and 2 h post-infection (Figure 5A).

To confirm the role of *L. salivarius* in the inhibition of PEDV infection, cells were first incubated with different concentrations of *L. salivarius* (0, 1, 10, and 100 MOI) for 2 h, and then incubated with PEDV for 1 or 2 h at a concentration of 0.1 MOI (Figure 5B). Viral RNA was consistently lower in *L. salivarius*-pretreated cells than in untreated cells at both 1 and 2 h post-infection. This incubated that *L. salivarius* has a protective effect on IPEC-J2 cells. When incubated with PEDV for 1 h, *L. salivarius*-treated cells were infected with significantly less PEDV various than untreated cells (*p* < 0.01). When IPEC-J2 cells were treated with 1 MOI *L. salivarius* for 2 h followed by a 2 h incubation with PEDV, PEDV infection was observed to decrease to the minimum level compared with other groups (*p* < 0.01). The results indicated that *L. salivarius* treatment at a concentration of 1 MOI exhibited a more robust activity against PEDV infection than treatments at other concentrations (Figure 5B). Therefore, we assumed that treatment with 1 MOI *L. salivarius* for 2 h, before 0.1 MOI PEDV inoculation to simulate the PEDV infection, in healthy piglets *in vitro*.

### *L. salivarius* Interferes With the Expression of Host Genes

**Figure 6:**
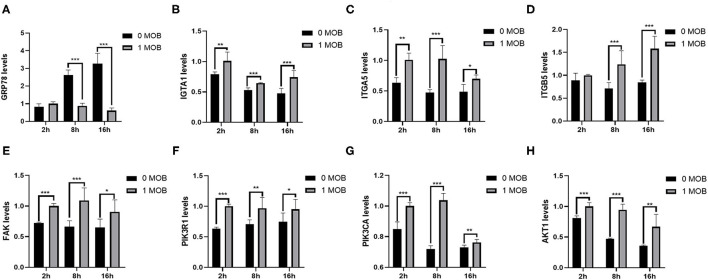
*Lactobacillus salivarius* interfere with the expression of genes in IPEC-J2 cells. **(A)** The mRNA expression of GRP78 in untreated and *L. salivarius-*treated groups was detected by qPCR at different times post-PEDV infection. **(B–H)** The mRNA expression of seven genes (*ITGA1, ITGA5, ITGB5, FAK, PIK3R1, PIK3CA* and *AKT1*) from the FAK/PI3K/Akt signaling pathway was examined in the two groups by qPCR at different times post-infection. The differences between the groups were analyzed using the *t*-test. Significant differences are marked with asterisks. ^*^: *p* < 0.05, ^**^: *p* < 0.01, ^***^: *p* < 0.001.

To investigate the effect of *L. salivarius* on host genes at the transcriptional level, the mRNA expression of some important genes inIPEC-J2 cells was quantified at 2, 8, and 16 h post PEDV-infection via qPCR. Glucose-regulating protein 78 (GRP78) is a chaperone protein of the endoplasmic reticulum (ER), and up-regulation of GRP78 expression is a sign of ER stress. To determine whether PEDV infection causes ER stress, the expression of *GRP78* in IPEC-J2 cells was evaluated at different time points post-PEDV infection by qPCR. Indeed, the expression level of *GRP78* increased gradually with the increase in viral infection time (Figure 6A). Furthermore, we examined whether pretreatment with *L. salivarius* would reduce ER stress. Compared with untreated cells, the 1 MOI *L. salivarius*-treated group showed a gradual decrease in the expression of *GRP78*, which was significantly lower than that in untreated cells at 8 and 16 h post-PEDV infection (Figure 6A).

The PI3K-Akt signaling pathway plays an important role in PEDV infection. The focal adhesion signaling pathway is also involved in the process of PEDV infection. Therefore, we quantified the mRNA expression levels of *ITGA1, ITGA5, ITGB5, FAK, PIK3R1, PIK3CA* and *AKT1* in IPEC-J2 at 2, 8, and 16 h post-PEDV infection (Figures 6B–H). The expression levels of *ITGA1, ITGA5, FAK, PIK3R1, PIK3CA* and *AKT1* were significantly higher in the 1 MOI *L. salivarius*-treated group, than in untreated cells at 2, 8, and 16 h post-PEDV infection. Although there was no significant difference in the expression of *ITGB5* between the two groups at 2 h, the expression levels of *ITGB5* were significantly higher in the 1 MOI *L. salivarius*-treated group than in untreated cells at 8 and 16 h post-PEDV infection. The results indicated that *L. salivarius* may prevent PEDV infection in IPEC-J2 cells by up-regulating the FAK/PI3K/Akt signaling pathway.

## Discussion

After birth, suckling piglets gradually establish an intestinal microbial ecosystem, which is unstable and sensitive to pathogenic bacteria (36). However, suckling piglets are most sensitive to PED in the first week of birth (37), and the mortality of PED is close to 100% for newborn piglets and 80% for suckling piglets, resulting in severe losses to the swine industry (38). Several studies have shown that the intestinal microbiota of healthy animals plays a crucial role in maintaining the normal function of the intestinal mucosal barrier, development of the host's innate immune system, and maturation of the immune system. The gut microbiota also promotes the production of antibacterial compounds, creates a microenvironment resistant to pathogens, and affects the performance of infectious diseases.

Many studies, including our previous studies on the cecum and colon, have found that the intestinal microbiota of piglets infected with PEDV exhibits significant changes at both the phylum and genus levels, which disrupts normal intestinal microhomeostasis. For example, the proportion of *Escherichia -Shigella, Enterococcus, Fusobacterium, Actinobacillus, Campylobacter* and *Virginiella* increases significantly in piglets infected with PEDV. In contrast, the proportion of bacteria that produce short-chain fatty acids declines (22–25). The current study provides an overview of the microbial composition in the jejunal content and the jejunal mucosa. Firmicutes and Proteobacteria were the dominant phyla in the gut, particularly having the top two abundances in the jejunum (23). In contrast, some previously published studies have identified Bacteroidetes, not Proteobacteria, as the second dominant phylum (21, 22, 39). Firmicutes and Bacteroidetes are the dominant phyla in fecal samples of piglets (40). Furthermore, the composition of the small intestine and large intestine significantly differs in pigs (41), indicating that jejunal samples are more suitable than fecal samples for functional studies aimed at understanding the mechanism of PEDV infection.

At the genus level, the infected groups had a greater abundance of *Fusobacterium* in their jejunum than the uninfected group; similar results have been reported previously (21, 22, 39). The abundance of *Fusobacterium* in the L group was higher than that in the H and U groups, which is consistent with the results of previous studies. The proportion of *Lactobacillus* was higher in the uninfected group than in the infected groups, which is consistent with similar findings reported in previous studies (22–25, 39). A study focusing on diarrheic neonatal piglets found that *Lactobacillus* was enriched in the healthy group but not in the diarrhea group (42). In addition, we found that the abundance of *Escherichia-Shigella* increased after PEDV infection. Members of *Escherichia-Shigella* are regarded as conditional pathogenic bacteria in pigs. Fluorescence *in situ* hybridization identified potentially pathogenic bacteria that are involved in neonatal porcine diarrhea. The increase in the abundance of *Escherichia-Shigella* during infection can be partly explained by the imbalanced nature of the gut microbiota and a decrease in the ability of the immune system to defend against pathogens.

Our findings show that the abundance of *L. salivarius* in the jejunal mucosa of infected piglets was significantly different from that in uninfected piglets, and this bacterium was not found in digest samples. *L. salivarius* has also been reported to have a potential role in resisting different types of bacterial infection, including enterotoxigenic *Escherichia coli* (43, 44), *Salmonella enterica* Serovar Typhimurium (45), *Helicobacter pylori* (46), and *Listeria monocytogenes* (47). Moreover, *L. salivarius* is an effective alternative to antibiotics, and has been used in human clinical trials. For example, neonatal sepsis caused by Group B *Streptococci* was reduced when pregnant women consumed *L. salivarius* CECT 9145 (48). Oral intake of *L. salivarius* PS7 over 6 months led to a significant reduction (84%) in the number of episodes of acute otitis media, compared to those individuals in the same population, during the 6 months preceding the probiotic intervention (49). In the animal industry, oral administration of *L. salivarius* before F4+ infection with enterotoxigenic *Escherichia coli* has been shown to reduce diarrhea (50). *Salmonella enteritidis* colonization and the levels of pre-inflammatory factors in broiler chickens were reduced after feeding with a *Lactobacillus*-based probiotic mixture containing *L. salivarius* (51). Our results show the potential role of *L. salivarius* in the defense against PEDV infection, which has not previously been reported. Furthermore, the results of *in vitro* infection experiments validated the reliability of our sequencing data.

To understand how *L. salivarius* contributes toporcine intestinal health and helps protect against PEDV infection, we conducted *in vitro* PEDV infection experiments using porcine intestinal epithelial cells. Previous studies have shown that the PEDV N protein localizes in the ER and that PEDV infection significantly increases the expression of GRP78, leading to ER stress (52, 53). Therefore, we quantified the expression of *GRP78* in IPEC-J2 cells and found that *GRP78* expression was significantly increased in PEDV-infected cells without *L. salivarius* pretreatment. In contrast, *GRP78* expression in cells pretreated with 1 MOI *L. salivarius* decreased over time, indicating that *L. salivarius* can inhibit the ER stress caused by PEDV infection. In addition, the PI3K-Akt signaling pathway plays an important role in host cell responses to coronaviruses (54). Recent studies have found that the PI3K/Akt signaling pathway is down-regulated in PEDV-infected IPEC-J2 cells (55, 56). Lin *et al*. found that PEDV suppresses protein synthesis in host cells by negatively regulating the PI3K-Akt/mTOR signaling pathway (55). *PIK3R1, PIK3AC* and *AKT1* are the key components of the PI3K-Akt signaling pathway. The results of the current study showed that the mRNA expression levels of *PIK3R1, PIK3AC* and *AKT1* in the *L. salivarius*-treated group were significantly higher than those in the untreated group at different time post-PEDV infection. Furthermore, the FAK pathway contributes to cell migration, adhesion, and survival. For example, FAK regulates many cellular processes that are essential for epithelial homeostasis and restitution (57). Furthermore, the FAK signaling pathway is involved in the process of PEDV infection (58). A recent study demonstrated that the absence of FAK leads to the reduction of AKT and the downstream mTOR pathway, while the inhibition of the FAK and mTOR pathways induces cell apoptosis (59). Thus, we quantified the expression of *ITGA1, ITGA5, ITGB5* and *FAK* in the two groups. Our results showed that the mRNA expression levels of these four genes were up-regulated in the *L. salivarius*-treated group. These results suggested that *L. salivarius* may promote IPEC-J2 cell resistance to PEDV infection by up-regulating FAK/PI3K/Akt signaling. Many studies have demonstrated the importance of these pathways in regulating cell survival (57, 60–62). Therefore, we speculate that the FAK/PI3K/Akt axis is involved in cell cytopathology, cell survival, and apoptosis through a variety of mechanisms after PEDV infection. Our results may provide the basis for further analyses of the mechanism whereby *L. salivarius* promotes piglet host resistance against PEDV infection.

This study used 16S rRNA sequencing to determine the microbiota profile of jejunal content and jejunal mucosa samples from PEDV-infected piglets. The results suggested that PEDV infection reduces the proportion of probiotic bacteria in both jejunal content and jejunal mucosa. A novel finding of the current study is that *L. salivarius* appears to be involved in host resistance to PEDV infection. Furthermore, we found that the FAK/PI3K/Akt axis plays an important role in antiviral regulation in IPEC-J2 cells. These findings contribute to a comprehensive understanding of the mechanism of PEDV infection in piglets, and will help develop future clinical intervention strategies against PEDV infection in the pig industry.

## Data Availability Statement

The datasets presented in this study can be found in online repositories. The names of the repository/repositories and accession number(s) can be found below: https://www.ncbi.nlm.nih.gov/, SRP334838.

## Ethics Statement

The animal study was reviewed and approved by Quality Supervision, Inspection, and Quarantine of the People's Republic of China (GB/T 17236–2008). The animal experimental proposals were approved by the Animal Welfare Committee of China Agricultural University (permit number: DK996).

## Author Contributions

LJ, XD, and QZ: conceptualization. WD, ZT, and LJ: methodology and data curation. WD: software and writing-original draft preparation. WD, ND, and YZ: resources. ZT and LJ: writing-review and editing and funding acquisition. WD and ZT: visualization. QZ and LJ: project administration. All authors have read and agreed to the published version of the manuscript.

## Funding

This work was supported by the China Agriculture Research System of MOF and MARA (CARS-35), the National Key Research and Development Program of China (2019YFE0106800), the Key Research and Development Project of Hainan Province (ZDYF2020094), and the Research Initiation Fund of Hainan University (KYQD(ZR)1920).

## Conflict of Interest

The authors declare that the research was conducted in the absence of any commercial or financial relationships that could be construed as a potential conflict of interest.

## Publisher's Note

All claims expressed in this article are solely those of the authors and do not necessarily represent those of their affiliated organizations, or those of the publisher, the editors and the reviewers. Any product that may be evaluated in this article, or claim that may be made by its manufacturer, is not guaranteed or endorsed by the publisher.

## Supplementary Material

Supplementary Figure 1 Quantification of viral copy numbers by qPCR. U group [uninfected samples, *n* = 8, −1.0904 ± 0.2029 log(copies)], L group [low viral load samples, *n* = 3, 0.3713 ± 0.4093 log(copies)] and H group [high viral load samples, *n* = 5, 3.0170 ± 0.522 log(copies)]. Low viral load and high viral load piglets are two groups of piglets suffering from diarrhea with significantly different copy numbers of PEDV (Kruskal–Wallis test, *p* < 0.01).

Supplementary Table 1 Alpha diversity indices in jejunal microbiota from uninfected and PEDV-infected piglets. HC, jejunal content microbiota in the high viral load group; LC, jejunal content microbiota in the low viral load group; UC, jejunal content microbiota in the uninfected group; HM, jejunal mucosa microbiota in the high viral load group; LM, jejunal mucosa microbiota in the low viral load group; UM, jejunal mucosa microbiota in the uninfected group; Shannon and Simpson indices were used to assess biodiversity; the species index shows the number of OTUs that were observed; the Good's coverage index was used to reflect the species coverage; Chao1 indices were used to estimate the number of OTUs and microbial richness; ^*^ denotes significant differences in alpha diversity indices between the groups.

## References

[1] Alonso C Goede DP Morrison RB Davies PR Rovira A Marthaler DG. Evidence of infectivity of airborne porcine epidemic diarrhea virus and detection of airborne viral RNA at long distances from infected herds. BioMed Central. (2014) 45:73. 10.1186/s13567-014-0073-z 25017790 PMC4347589

[2] Huang YW Dickerman AW Pineyro P Li L Fang L Kiehne R. Origin, evolution, and genotyping of emergent porcine epidemic diarrhea virus strains in the United States. MBio. (2013) 4:e00737–e00713. 10.1128/mBio.00737-13 24129257 PMC3812708

[3] Chen YF Zhang ZB Li J Gao YY Zhou L Ge XN. Porcine epidemic diarrhea virus S1 protein is the critical inducer of apoptosis. Virol J. (2018) 15:170. 10.1186/s12985-018-1078-4 30404647 PMC6222994

[4] Wang D Fang L Xiao S. Porcine epidemic diarrhea in China. Virus Res. (2016) 226:7–13. 10.1016/j.virusres.2016.05.026 27261169 PMC7114554

[5] Jung K Saif LJ. Porcine epidemic diarrhea virus infection: Etiology, epidemiology, pathogenesis immunoprophylaxis. Vet J. (2015) 204:134–43. 10.1016/j.tvjl.2015.02.017 25841898 PMC7110711

[6] Park S Kim S Song D Park B. Novel porcine epidemic diarrhea virus variant with large genomic deletion, South Korea. Emerg Infect Dis. (2014) 20:2089–92. 10.3201/eid2012.131642 25424875 PMC4257805

[7] Kim YK Lim SI Lim JA Cho IS Park EH Le VP. A novel strain of porcine epidemic diarrhea virus in Vietnamese pigs. Arch Virol. (2015) 160:1573–7. 10.1007/s00705-015-2411-5 25864174

[8] Ojkic D Hazlett M Fairles J Marom A Slavic D Maxie G. The first case of porcine epidemic diarrhea in Canada. Can Vet J. (2015) 56:149–52. 25694663 PMC4298265

[9] Pensaert MB de Bouck P. A new coronavirus-like particle associated with diarrhea in swine. Arch Virol. (1978) 58:243–7. 10.1007/BF01317606 83132 PMC7086830

[10] Li Z Chen F Yuan Y Zeng X Wei Z Zhu L. Sequence and phylogenetic analysis of nucleocapsid genes of porcine epidemic diarrhea virus (PEDV) strains in China. Arch Virol. (2013) 158:1267–73. 10.1007/s00705-012-1592-4 23389550 PMC3668129

[11] Wang E Guo D Li C Wei S Wang Z Liu Q. Molecular Characterization of the ORF3 and S1 Genes of Porcine Epidemic Diarrhea Virus Non S-INDEL Strains in Seven Regions of China, 2015. PLoS ONE. (2016) 11:e0160561. 10.1371/journal.pone.0160561 27494026 PMC4975444

[12] Guinane CM Cotter PD. Role of the gut microbiota in health and chronic gastrointestinal disease: understanding a hidden metabolic organ. Therap Adv Gastroenterol. (2013) 6:295–308. 10.1177/1756283X13482996 23814609 PMC3667473

[13] Kamada N Chen GY Inohara N Nunez G. Control of pathogens and pathobionts by the gut microbiota. Nat Immunol. (2013) 14:685–90. 10.1038/ni.2608 23778796 PMC4083503

[14] Leshem A Liwinski T Elinav E. Immune-Microbiota Interplay and Colonization Resistance in Infection. Mol Cell. (2020) 78:597–613. 10.1016/j.molcel.2020.03.001 32208169

[15] Littman DR Pamer EG. Role of the commensal microbiota in normal and pathogenic host immune responses. Cell Host Microbe. (2011) 10:311–23. 10.1016/j.chom.2011.10.004 22018232 PMC3202012

[16] Cai R Cheng C Chen J Xu X Ding C Gu B. Interactions of commensal and pathogenic microorganisms with the mucus layer in the colon. Gut Microbes. (2020) 11:680–90. 10.1080/19490976.2020.1735606 32223365 PMC7524288

[17] Hergott CB Roche AM Tamashiro E Clarke TB Bailey AG Laughlin A. Peptidoglycan from the gut microbiota governs the lifespan of circulating phagocytes at homeostasis. Blood. (2016) 127:2460–71. 10.1182/blood-2015-10-675173 26989200 PMC4874226

[18] Honda K Littman DR. The microbiota in adaptive immune homeostasis and disease. Nature. (2016) 535:75–84. 10.1038/nature18848 27383982

[19] Thaiss CA Zmora N Levy M Elinav E. The microbiome and innate immunity. Nature. (2016) 535:65–74. 10.1038/nature18847 27383981

[20] Koh HW Kim MS Lee JS Kim H Park SJ. Changes in the Swine Gut Microbiota in Response to Porcine Epidemic Diarrhea Infection. Microbes Environ. (2015) 30:284–7. 10.1264/jsme2.ME15046 26212519 PMC4567570

[21] Liu S Zhao L Zhai Z Zhao W Ding J Dai R. Porcine Epidemic Diarrhea Virus Infection Induced the Unbalance of Gut Microbiota in Piglets. Curr Microbiol. (2015) 71:643–9. 10.1007/s00284-015-0895-6 26319658

[22] Song D Peng Q Chen Y Zhou X Zhang F Li A. Altered Gut Microbiota Profiles in Sows and Neonatal Piglets Associated with Porcine Epidemic Diarrhea Virus Infection. Sci Rep. (2017) 7:17439. 10.1038/s41598-017-17830-z 29234140 PMC5727058

[23] Huang MZ Wang SY Wang H Cui DA Yang YJ Liu XW. Differences in the intestinal microbiota between uninfected piglets and piglets infected with porcine epidemic diarrhea virus. Plos ONE. (2018) 13:e0192992. 10.1371/journal.pone.0192992 29447243 PMC5814011

[24] Tan Z Dong W Ding Y Ding X Zhang Q Jiang L. Porcine epidemic diarrhea altered colonic microbiota communities in suckling piglets. Genes (Basel). (2019) 11:44. 10.3390/genes11010044 31905830 PMC7016528

[25] Tan Z Dong WT Ding YQ Ding XD Zhang Q Jiang L. Changes in cecal microbiota community of suckling piglets infected with porcine epidemic diarrhea virus. PLoS ONE. (2019) 14:e0219868. 10.1371/journal.pone.0219868 31310635 PMC6634403

[26] Zhou X Zhang T Song D Huang T Peng Q Chen Y. Comparison and evaluation of conventional RT-PCR, SYBR green I and TaqMan real-time RT-PCR assays for the detection of porcine epidemic diarrhea virus. Mol Cell Probes. (2017) 33:36–41. 10.1016/j.mcp.2017.02.002 28188840

[27] Kozich JJ Westcott SL Baxter NT Highlander SK Schloss PD. Development of a dual-index sequencing strategy and curation pipeline for analyzing amplicon sequence data on the MiSeq Illumina sequencing platform. Appl Environ Microbiol. (2013) 79:5112–20. 10.1128/AEM.01043-13 23793624 PMC3753973

[28] Caporaso JG Kuczynski J Stombaugh J Bittinger K Bushman FD Costello EK. QIIME allows analysis of high-throughput community sequencing data. Nat Methods. (2010) 7:335–6. 10.1038/nmeth.f.303 20383131 PMC3156573

[29] Magoc T Salzberg SL. FLASH: fast length adjustment of short reads to improve genome assemblies. Bioinformatics. (2011) 27:2957–63. 10.1093/bioinformatics/btr507 21903629 PMC3198573

[30] Edgar RC Haas BJ Clemente JC Quince C Knight R. UCHIME improves sensitivity and speed of chimera detection. Bioinformatics. (2011) 27:2194–200. 10.1093/bioinformatics/btr381 21700674 PMC3150044

[31] Edgar RC. UPARSE: highly accurate OTU sequences from microbial amplicon reads. Nat Methods. (2013) 10:996–8. 10.1038/nmeth.2604 23955772

[32] Quast C Pruesse E Yilmaz P Gerken J Schweer T Yarza P. The SILVA ribosomal RNA gene database project: improved data processing and web-based tools. Nucleic Acids Res. (2013) 41:D590–596. 10.1093/nar/gks1219 23193283 PMC3531112

[33] Schloss PD Westcott SL Ryabin T Hall JR Hartmann M Hollister EB. Introducing mothur: open-source, platform-independent, community-supported software for describing and comparing microbial communities. Appl Environ Microbiol. (2009) 75:7537–41. 10.1128/AEM.01541-09 19801464 PMC2786419

[34] Lozupone C Lladser ME Knights D Stombaugh J Knight R. UniFrac: an effective distance metric for microbial community comparison. ISME J. (2011) 5:169–72. 10.1038/ismej.2010.133 20827291 PMC3105689

[35] Segata N Izard J Waldron L Gevers D Miropolsky L Garrett WS. Metagenomic biomarker discovery and explanation. Genome Biol. (2011) 12:R60. 10.1186/gb-2011-12-6-r60 21702898 PMC3218848

[36] Ivanov II Littman DR. Modulation of immune homeostasis by commensal bacteria. Curr Opin Microbiol. (2011) 14:106–14. 10.1016/j.mib.2010.12.003 21215684 PMC3123735

[37] Song D Park B. Porcine epidemic diarrhoea virus: a comprehensive review of molecular epidemiology, diagnosis, and vaccines. Virus Genes. (2012) 44:167–75. 10.1007/s11262-012-0713-1 22270324 PMC7089188

[38] Beth M. Deadly pig virus slips through US borders. Nature. (2013) 499:388. 10.1038/499388a 23887408

[39] Huang A Cai R Wang Q Shi L Li C Yan H. Dynamic Change of Gut Microbiota During Porcine Epidemic Diarrhea Virus Infection in Suckling Piglets. Front Microbiol. (2019) 10:322. 10.3389/fmicb.2019.00322 30858839 PMC6397872

[40] Piccolo BD Mercer KE Bhattacharyya S Bowlin AK Saraf MK Pack L. Early Postnatal Diets Affect the Bioregional Small Intestine Microbiome and Ileal Metabolome in Neonatal Pigs. J Nutr. (2017) 147:1499–509. 10.3945/jn.117.252767 28659406

[41] Liu Y Zheng Z Yu L Wu S Sun L Wu S. Examination of the temporal and spatial dynamics of the gut microbiome in newborn piglets reveals distinct microbial communities in six intestinal segments. Sci Rep. (2019) 9:3453. 10.1038/s41598-019-40235-z 30837612 PMC6400902

[42] Yang Q Huang X Zhao S Sun W Yan Z Wang P. Structure and Function of the Fecal Microbiota in Diarrheic Neonatal Piglets. Front Microbiol. (2017) 8:502. 10.3389/fmicb.2017.00502 28392784 PMC5364137

[43] Tsai CC Lin PP Hsieh YM. Three Lactobacillus strains from healthy infant stool inhibit enterotoxigenic Escherichia coli grown *in vitro*. Anaerobe. (2008) 14:61–7. 10.1016/j.anaerobe.2007.11.003 18182312

[44] Yeo S Lee S Park H Shin H Holzapfel W Huh CS. Development of putative probiotics as feed additives: validation in a porcine-specific gastrointestinal tract model. Appl Microbiol Biotechnol. (2016) 100:10043–54. 10.1007/s00253-016-7812-1 27633101 PMC5102953

[45] Casey PG Gardiner GE Casey G Bradshaw B Lawlor PG Lynch PB. A five-strain probiotic combination reduces pathogen shedding and alleviates disease signs in pigs challenged with Salmonella enterica Serovar Typhimurium. Appl Environ Microbiol. (2007) 73:1858–63. 10.1128/AEM.01840-06 17261517 PMC1828830

[46] Ryan KA O'Hara AM van Pijkeren JP Douillard FP O'Toole PW. Lactobacillus salivarius modulates cytokine induction and virulence factor gene expression in Helicobacter pylori. J Med Microbiol 58. (2009) 996–1005. 10.1099/jmm.0.009407-0 19528183

[47] Corr SC Li Y Riedel CU O'Toole PW Hill C Gahan CGM. Bacteriocin production as a mechanism for the antiinfective activity of Lactobacillus salivarius UCC118. Proc Natl Acad Sci U S A. (2007) 104:7617–21. 10.1073/pnas.0700440104 17456596 PMC1863472

[48] Martin V Cardenas N Ocana S Marin M Arroyo R Beltran D. Rectal and vaginal eradication of streptococcus agalactiae (GBS) in pregnant women by using lactobacillus salivarius CECT 9145, a target-specific probiotic strain. Nutrients. (2019) 11:810. 10.3390/nu11040810 30974819 PMC6521265

[49] Cardenas N Martin V Arroyo R Lopez M Carrera M Badiola C. Prevention of recurrent acute otitis media in children through the use of lactobacillus salivarius PS7, a target-specific probiotic strain. Nutrients. (2019) 11:376. 10.3390/nu11020376 30759799 PMC6413216

[50] Sayan H Assavacheep P Angkanaporn K Assavacheep A. Effect of Lactobacillus salivarius on growth performance, diarrhea incidence, fecal bacterial population and intestinal morphology of suckling pigs challenged with F4(+) enterotoxigenic Escherichia coli. Asian-australas J Anim Sci. (2018) 31:1308–14. 10.5713/ajas.17.0746 29642683 PMC6043459

[51] Penha RAC Diaz SJA Fernando FS Chang YF Andreatti RL Berchieri A. Immunomodulatory activity and control of Salmonella Enteritidis colonization in the intestinal tract of chickens by Lactobacillus based probiotic. Vet Immunol Immunopathol. (2015) 167:64–9. 10.1016/j.vetimm.2015.06.006 26099807

[52] Xu X Zhang H Zhang Q Huang Y Dong J Liang Y. Porcine epidemic diarrhea virus N protein prolongs S-phase cell cycle, induces endoplasmic reticulum stress, and up-regulates interleukin-8 expression. Vet Microbiol. (2013) 164:212–21. 10.1016/j.vetmic.2013.01.034 23562137 PMC7117426

[53] Pei S Jian J Lixiang W Jingjing W Hongchao Z Qi Z. Porcine epidemic diarrhea virus infections induce autophagy in Vero cells via ROS-dependent endoplasmic reticulum stress through PERK and IRE1 pathways. Vet Microbiol. (2021) 253. 10.1016/j.vetmic.2020.108959 33360915

[54] Mizutani T Fukushi S Saijo M Kurane I Morikawa JNK S and PI3k/Akt signaling pathways are required for establishing persistent SARS-CoV infection in Vero E6 cells. Biochim Biophys Acta. (2005) 1741:4–10. 10.1016/j.bbadis.2005.04.004 15916886 PMC7125767

[55] Lin H Li B Chen L Ma Z He K Fan H. Differential Protein Analysis of IPEC-J2 Cells Infected with Porcine Epidemic Diarrhea Virus Pandemic and Classical Strains Elucidates the Pathogenesis of Infection. J Proteome Res. (2017) 16:2113–20. 10.1021/acs.jproteome.6b00957 28506058

[56] Shen X Yin L Pan X Zhao R Zhang D. Porcine epidemic diarrhea virus infection blocks cell cycle and induces apoptosis in pig intestinal epithelial cells. Microb Pathog. (2020) 147:104378. 10.1016/j.micpath.2020.104378 32653434 PMC7347497

[57] Owen KA Abshire MY Tilghman RW Casanova JE Bouton AH. FAK regulates intestinal epithelial cell survival and proliferation during mucosal wound healing. PLoS ONE. (2011) 6:e23123. 10.1371/journal.pone.0023123 21887232 PMC3160839

[58] Sun D Shi H Guo D Chen J Shi D Zhu Q. Analysis of protein expression changes of the Vero E6 cells infected with classic PEDV strain CV777 by using quantitative proteomic technique. J Virol Methods. (2015) 218:27–39. 10.1016/j.jviromet.2015.03.002 25783682 PMC7113725

[59] Paul R Luo M Mo X Lu J Yeo SK Guan JL. FAK activates AKT-mTOR signaling to promote the growth and progression of MMTV-Wnt1-driven basal-like mammary tumors. Breast Cancer Res. (2020) 22:59. 10.1186/s13058-020-01298-3 32493400 PMC7268629

[60] Mitra SK Schlaepfer DD. Integrin-regulated FAK-Src signaling in normal and cancer cells. Curr Opin Cell Biol. (2006) 18:516–23. 10.1016/j.ceb.2006.08.011 16919435

[61] Yu JS Cui W. Proliferation, survival and metabolism: the role of PI3K/AKT/mTOR signalling in pluripotency and cell fate determination. Development. (2016) 143:3050–60. 10.1242/dev.137075 27578176

[62] Lin H Li B Liu M Zhou H He K Fan H. Nonstructural protein 6 of porcine epidemic diarrhea virus induces autophagy to promote viral replication via the PI3K/Akt/mTOR axis. Vet Microbiol. (2020) 244:108684. 10.1016/j.vetmic.2020.108684 32402351 PMC7165116

