# Supplementary figures and images for: Alterations of Suckling Piglet Jejunal Microbiota Due to Infection With Porcine Epidemic Diarrhea Virus and Protection Against Infection by Lactobacillus salivarius
Source: Front Vet Sci. 2021 Dec 9;8:771411. doi: 10.3389/fvets.2021.771411 (PMC8695681; doi:10.3389/fvets.2021.771411)

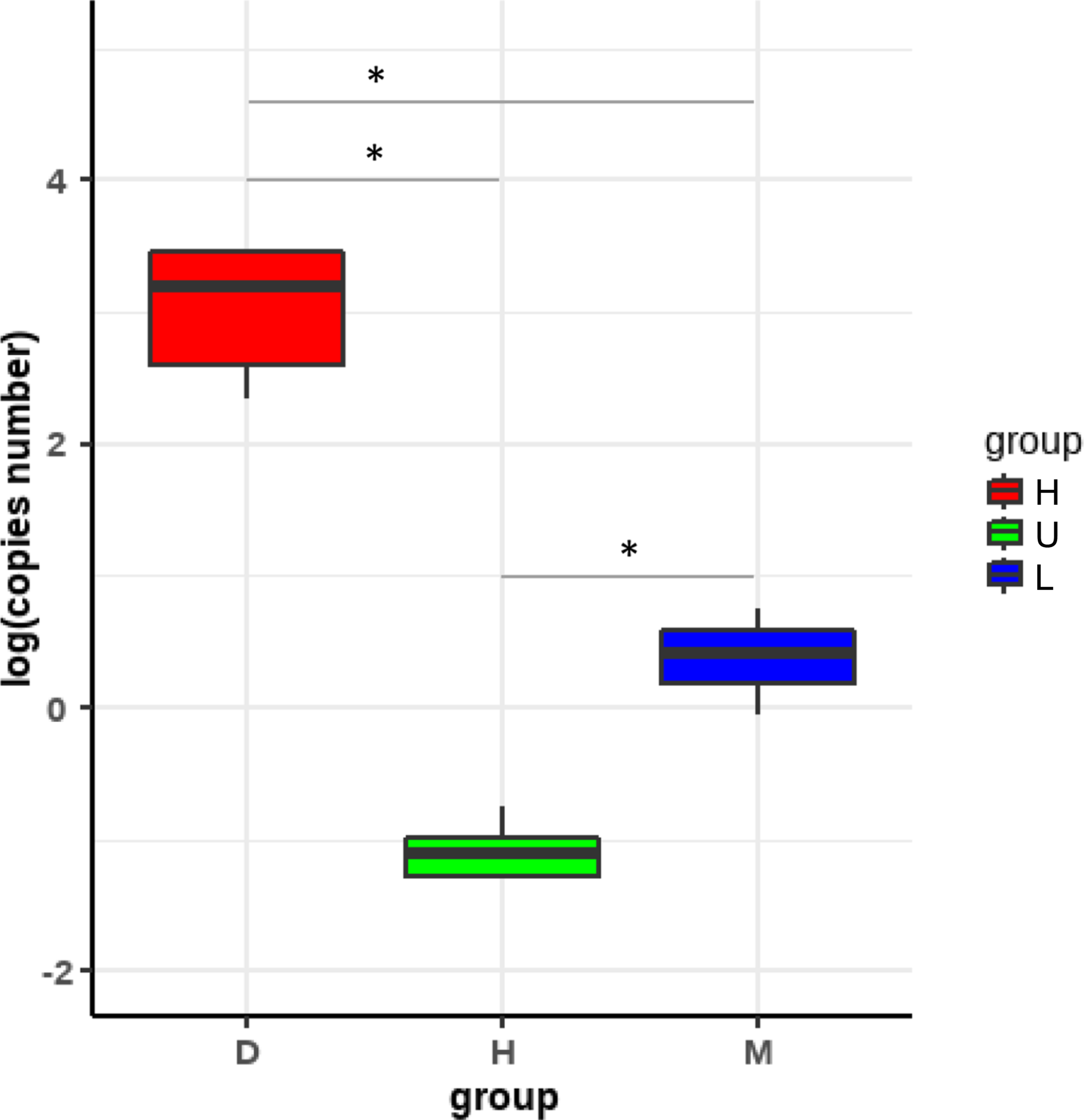

Supplement: Supplementary Figure 1 — Quantification of viral copy numbers by qPCR. U group [uninfected samples, n = 8, −1.0904 ± 0.2029 log(copies)], L group [low viral load samples, n = 3, 0.3713 ± 0.4093 log(copies)] and H group [high viral load samples, n = 5, 3.0170 ± 0.522 log(copies)]. Low viral load and high viral load piglets are two groups of piglets suffering from diarrhea with significantly different copy numbers of PEDV (Kruskal–Wallis test, p < 0.01). [file Image_1.TIF]
