# Supplementary material for: Alterations of Suckling Piglet Jejunal Microbiota Due to Infection With Porcine Epidemic Diarrhea Virus and Protection Against Infection by Lactobacillus salivarius
Source: Front Vet Sci. 2021 Dec 9;8:771411. doi: 10.3389/fvets.2021.771411 (PMC8695681; doi:10.3389/fvets.2021.771411)
Supplement: Supplementary Table 1 — Alpha diversity indices in jejunal microbiota from uninfected and PEDV-infected piglets. HC, jejunal content microbiota in the high viral load group; LC, jejunal content microbiota in the low viral load group; UC, jejunal content microbiota in the uninfected group; HM, jejunal mucosa microbiota in the high viral load group; LM, jejunal mucosa microbiota in the low viral load group; UM, jejunal mucosa microbiota in the uninfected group; Shannon and Simpson indices were used to assess biodiversity; the species index shows the number of OTUs that were observed; the Good's coverage index was used to reflect the species coverage; Chao1 indices were used to estimate the number of OTUs and microbial richness; * denotes significant differences in alpha diversity indices between the groups. [file Table_1.DOCX]

**Supplementary Table 1. Alpha diversity indexes of jejunal microbiota between uninfected and PEDV infected piglets.**

| Group | P-value |  | Simpson | Shannon | Observed_species | Goods_coverage | Chao1 |
| --- | --- | --- | --- | --- | --- | --- | --- |
| HC |  |  | 0.592±0.206 | 2.391±1.112 | 182±51 | 0.9996±0.0001 | 186±49 |
| LC |  |  | 0.897±0.086 | 4.594±1.470 | 285±78 | 0.9995±0.0001 | 286±79 |
| UC |  |  | 0.838±0.208 | 4.174±1.178 | 206±60 | 0.9997±0.0002 | 209±61 |
| HC-LC | wilcox |  | 0.0216^*^ | 0.0441^*^ | 0.072 | 0.716 | 0.081 |
| LC-UC | wilcox |  | 0.541 | 0.487 | 0.122 | 0.103 | 0.115 |
| HC-UC | wilcox |  | 0.0221* | 0.066 | 0.592 | 0.132 | 0.681 |
| HM |  |  | 0.747±0.227 | 3.867±1.832 | 665±515 | 0.996±0.0034 | 832±672 |
| LM |  |  | 0.875±0.139 | 4.934±1.963 | 632±398 | 0.997±0.0026 | 803±578 |
| UM |  |  | 0.871±0.238 | 5.848±1.868 | 1398±786 | 0.992±0.0058 | 1831±1146 |
| HM-LM | wilcox |  | 0.377 | 0.385 | 0.919 | 0.773 | 0.904 |
| LM-UM | wilcox |  | 0.619 | 0.487 | 0.150 | 0.189 | 0.140 |
| HM-UM | wilcox |  | 0.099 | 0.067 | 0.073 | 0.226 | 0.111 |

HC, jejunal content microbiota of high viral load piglets; LC, jejunal content microbiota of low viral load piglets; UC, jejunal content microbiota of uninfected piglets; HM, jejunal mucosa microbiota of high viral load piglets; LM, jejunal mucosa microbiota of low viral load piglets; UM, jejunal mucosa microbiota of uninfected piglets; Shannon and Simpson indices were used to assess biodiversity; the observed species index shows the number of OTUs actually observed; the goods coverage index was used to reflect the species coverage;Chao1 indices were used to estimate the number of OTUs and microbial richness; *denote significant differences in alpha diversity indices between groups.
